# The presence and relative abundance of salivary *Fusobacterium nucleatum* are not associated with colorectal cancer: a systematic review and meta-analysis

**DOI:** 10.1038/s41598-025-07465-w

**Published:** 2025-07-10

**Authors:** Ellay Gutmacher, Bálint Zsombor Sárai, Petrana Martineková, Szilvia Kiss-Dala, Gergely Agócs, Péter Hegyi, Andrea Bródy, Ákos Zsembery

**Affiliations:** 1 Centre for Translational Medicine, Semmelweis University, Budapest, Hungary; 2 Department of Public Dental Health, Semmelweis University, Budapest, Hungary; 3 Department of Biophysics and Radiation Biology, Semmelweis University, Budapest, Hungary; 4 Institute of Pancreatic Diseases, Semmelweis University, Budapest, Hungary; 5 Institute for Translational Medicine, Medical School, University of Pécs, Pécs, Hungary; 6 Department of Oral Diagnostics, Semmelweis University, Budapest, Hungary; 7 Department of Oral Biology, Faculty of Dentistry, Semmelweis University, Budapest, Hungary

**Keywords:** Colorectal cancer, Fusobacterium nucleatum, Saliva, Biomarker, Non-invasive screening, Meta-analysis, Cancer imaging, Bacteriology, Diagnostic markers

## Abstract

Colorectal cancer (CRC) is the third most common cancer worldwide and the second leading cause of cancer-related deaths. Early detection through non-invasive methods is critical for improving patient outcomes. This study investigates the association between the presence and relative abundance of Fusobacterium nucleatum (Fn) in saliva and CRC, evaluating its potential as a non-invasive biomarker. A systematic search was performed in MEDLINE, Embase, CENTRAL, and Scopus databases on November 25, 2023. Studies analyzing Fn in salivary samples from adults with CRC, colorectal polyps (CRP), or healthy individuals were included. Statistical analyses were performed using random-effects models to calculate pooled odds ratios (OR) and mean differences (MD) with 95% confidence intervals (CI). The risk of bias was assessed using the Quality in Prognosis Studies (QUIPS) tool. Of the 14,200 studies identified, twelve were included in our systematic review. Of these, eight were analyzed by meta-analysis. The results indicated no significant difference in the presence (OR 1.40; 95% CI [0.77; 2.54]; *I*^2^ = 0% [0; 71%], *p* = 0.215) or relative abundance (MD -0.01; 95% CI [-0.13; 0.11]; *I*^2^ = 25% [0; 69%], *p* = 0.851) of salivary Fn among CRC patients, compared to a combined group of CRP and healthy controls. Our findings suggest that the presence and relative abundance of salivary Fn are not associated with CRC.

**Supplementary Information:**

The online version contains supplementary material available at 10.1038/s41598-025-07465-w.

## Introduction

Colorectal cancer (CRC) ranks as the third most prevalent cancer globally, accounting for roughly 10% of all cancer cases. It is also the second leading cause of cancer-related deaths worldwide^1^. Early detection and appropriate treatment are essential for enhancing patient survival and lowering mortality rates^2^. Colonoscopy prevents early CRC by detecting and removing colorectal polyps^3^. However, this procedure is invasive, necessitating bowel preparation and intravenous analgesia, and carries risks such as bowel perforation, hemorrhage, and cardiovascular events^4^. Pain and fear associated with colonoscopy can discourage patients from participating in screenings^5^. Furthermore, geographic barriers, such as the lack of nearby healthcare facilities and insufficient availability of specialists in remote communities, limit colonoscopy access, leading to late diagnoses^6^.

Fusobacterium nucleatum (Fn) is an oral pathogenic bacterium associated with periodontitis^7^. It has been found to accelerate carcinogenesis in various types of cancer, including pancreatic, breast, and CRC^8^. Fn is involved in both the occurrence and metastasis of CRC through mechanisms such as regulating immune response, virulence factors, oncogenic microRNAs, and DNA damage^9^. An increased abundance of Fn has been observed in the colorectal tissues of patients with CRC and polyposis, with higher bacterial abundance correlating with lower survival rates^10^. Whole genome sequencing of paired oral and CRC Fn isolates demonstrated that oral fusobacteria could translocate to CRC by descending the digestive tract or through the bloodstream during transient bacteremia caused by activities such as chewing, daily hygiene, or dental procedures^11^. Interestingly, Zepeda-Rivera et al. found that the CRC tumor-isolated strains predominantly belong to Fn subspecies animalis (Fna). A specific clade of Fna, such as Fna C2, has been shown to alter the metabolic profile of the tumor microenvironment, promoting adenoma formation and cancer progression^12^.

Recent studies have suggested that Fn may serve as a potential biomarker for CRC^13,14^. Moreover, salivary Fn levels have shown promise as a non-invasive diagnostic tool^15^. One study has demonstrated that salivary Fn detection can differentiate CRC patients from healthy individuals with significant accuracy^16^. Saliva, an easily accessible biofluid, presents a promising medium for detecting CRC. Non-invasive diagnostic methods, such as detecting Fn in saliva, can significantly improve screening participation rates. Furthermore, understanding the diagnostic potential of salivary Fn may lead to the development of accessible and cost-effective screening tools, particularly in regions with limited access to colonoscopy. Therefore, this systematic review and meta-analysis aimed to explore the association between salivary Fn and CRC.

## Results

### Search and selection

**Fig. 1:**
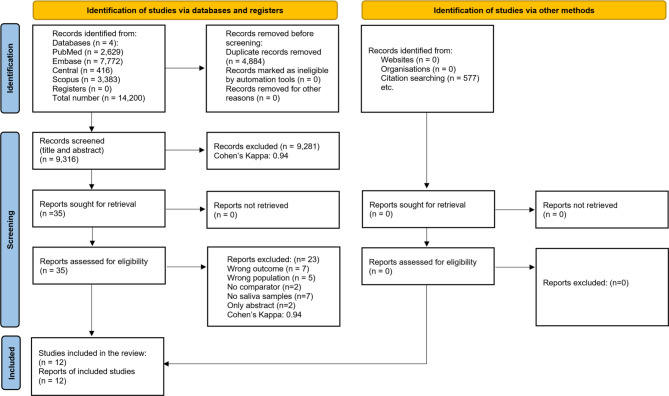
PRISMA flowchart of the screening and selection process.

Our search identified 14,200 studies. After duplicate removal and title and abstract selection (Cohen’s Kappa 0.94), 35 eligible articles remained for full-text analysis (Cohen’s Kappa 0.94). Of these, 23 articles were excluded due to overlapping populations (*n* = 5), no comparator (*n* = 2), different outcomes (*n* = 7), lack of salivary samples (*n* = 7), or abstract only (*n* = 2). Our systematic review and meta-analysis included 12 studies, 8 of which were suitable for a quantitative analysis. The selection process is summarized in Fig. 1.

### Baseline characteristics of included studies

The 12 studies included 901 CRC patients, 203 CRP patients, and 1,004 healthy individuals from Canada^17,18^Japan^19,20^Turkey^21^Italy^22,23^Vietnam^24^China^2,25,26^and the USA^27^. ‘Healthy individuals’ refers to individuals without CRC or CRP. Two studies were bicentric^21,23^and the others were single-center. The studies used non-quantitative polymerase chain reaction (PCR), quantitative PCR (qPCR), next-generation sequencing (NGS), 16S rRNA techniques or a combination of them to detect the presence and relative abundance of salivary Fn. Eight studies excluded individuals who consumed antibiotics either one/three months^17,19,22,25,26^, or one week^21,27,28^ prior to sampling, while four studies^2,18,23,24^ did not report data regarding the use of antibiotics. The baseline characteristics of the included studies are detailed in Table 1.

**Table 1:** Baseline characteristics of included studies.

Study	Region	Cases	Controls	°*N* of cases (% female)	°*N* of controls (% female)	Mean age ± SD (cases)`	Mean age ± SD (controls)	Exclusion based on AB use prior sampling
Guven 2019^21^	Turkey	CRC	Healthy	71 (35.2)	77 (51.9)	59	56	1 month
Janati 2022^17^	Canada	CRP + CRC	Healthy	22 (18)	21 (52)	63.9 ± 9.6	60.4 ± 9.1	3 months
Kageyama 2019^19^	Japan	CRC	Healthy	24 (33.3)	118 (29)	65.9 ± 10.9	66.4 ± 10.3	1 month
Limin Zhang 2023^26^	China	CRP	Healthy	33 (39.4)	22 (59.1)	59.12 ± 9.82	61.45 ± 6.93	3 months
Nearing (ATP cohort) 2023^18^	Canada	CRC	Healthy	22 (50)	22 (50)	60 ± 10	60 ± 9.99	Not reported
Nearing (PATH cohort retrospective) 2023^18^	Canada	CRC	Healthy	11 (5)	47 (53)	59.5 ± 10.2	56.9 ± 9.21	
Nearing (PATH cohort prospective) 2023^18^	Canada	CRC	Healthy	10 (70)	10 (70)	60.4 ± 7.88	60.7 ± 8.25	
Russo 2018^22^	Italy	CRC	Healthy	10 (60)	10 (40)	79 [77–84] ^a^	79 [73–84] ^a^	3 months
Russo 2023^23^	Italy	CRC	CRP	46 (28.3)	15 (53.33)	70.8	62.2	Not reported
Tran 2022^24^	Vietnam	CRC	CRP	43 (38)	21 (24)	64 [54–69] ^a^	60 [53–66] ^a^	Not reported
Uchino 2021^28^	Japan	CRC	Healthy	52 (36.5)	51 (49)	68.52 ± 10.6	54.49 ± 10.6	1 week
Wang 2021^25^	China	CRC	Healthy	142 (40.9)	95 (58)	65.07 ± 10.27	51.16 ± 10.75	1 month
Xin Zhang Training cohort 2022^2^	China	CRC	Healthy	207 (46.4)	41(43.9)	63 [48–71] ^a^	58 [45–67] ^a^	Not reported
	China	CRA	Healthy	43 (41.9)		56 [45–65] ^a^		
	China	HP	Healthy	33 (45.5)		57 [50–68] ^a^		
Xin Zhang Test cohort 2022^2^	China	CRC	Healthy	30 (43.3)	29 (37.93)	56 [36–66] ^a^	58 [46–65] ^a^	
	China	CRA	Healthy	17 (41.2)		54 [45–63] ^a^		
	China	HP	Healthy	21 (38.1)		56 [46–68] ^a^		
Yang 2018^27^	USA	CRC	Healthy	231 (59.7)	461 (59.9)	40–49 (22.0%)^b^	40–49 (24.1%)^b^	1 week
						50–59 (41.6%)^b^	50–59 (40.3%)^b^	
						60–69 (26.4%)^b^	60–69 (25.2%)^b^	
						70–79 (10%)^b^	70–79 (10.4%)b	

Study	Diagnostic procedure	Time point of saliva collection	DNA extraction kit	Bacterial analysis method				
Guven 2019^21^	Colonoscopy	Not reported	GeneMATRIX Swab-Extract DNA Purification Kit	qPCR				
Janati 2022^17^	Colonoscopy	Not reported	QIAamp DNA Mini Kit	qPCR				
Kageyama 2019^19^	Not reported	Not reported	bead-beating method	PCR + NGS + 16 S rRNA				
Limin Zhang 2023^26^	Colonoscopy	Not reported	Mag-Bind Blood & Tissue DNA HDQ 96 Kit	PCR + NGS + 16 S rRNA				
Nearing (ATP cohort) 2023^18^	Not reported	2.98 years after diagnosis (median)	DNA Genotek PrepIT PT-LP2 kit	PCR + NGS + 16 S rRNA				
Nearing (PATH cohort retrospective) 2023^18^	Not reported	5 years after diagnosis (median)	QIAamp 96 PowerFecal QIAcube HT kit					
Nearing (PATH cohort prospective) 2023^18^	Not reported	3 years after diagnosis (median)						
Russo 2018^22^	Colonoscopy	Day prior to surgery	PowerLyzer PowerSoil DNA Isolation Kit	qPCR + PCR+ NGS + 16 S rRNA				
Russo 2023^23^	Colonoscopy	Day of surgery	DNeasy PowerLyzer Power-Soil Kit	PCR + NGS + 16 S rRNA				
Tran 2022	Colonoscopy	3 h prior operation	ReliaPrep Blood gDNA Miniprep	PCR + NGS + 16 S rRNA				
Uchino 2021^28^	Not reported	Prior start of treatment	GENE STAR PI-480 automated DNA isolation system	PCR + qPCR + NGS + 16 S rRNA				
Wang 2021^25^	Colonoscopy	Prior surgery	QIAamp DNA mini kit	qPCR				
Xin Zhang Training cohort 2022^2^	Colonoscopy	Prior surgery	QIAamp DNA Mini Kit	qPCR + NGS				
	Colonoscopy							
	Colonoscopy							
Xin Zhang Test cohort 2022^2^	Colonoscopy	Not reported						
	Colonoscopy							
	Colonoscopy							
Yang 2018^27^	Not reported	Not reported	Qiagen’s QIAmp DNA kit	PCR + NGS + 16 S rRNA				

*AB* antibiotics, *CRA* colorectal adenoma, *CRC* colorectal cancer, *CRP* colorectal polyp, *DNA* deoxyribonucleic acid, *HP* hyperplastic polyp, *NGS* next-generation sequencing, *N* number, *PCR* polymerase chain reaction, *qPCR* quantitative PCR, *SD* standard deviation. ^a^Data expressed as median [interquartile range]; ^b^Data expressed as range of ages (% of individuals).

**Fig. 2:**
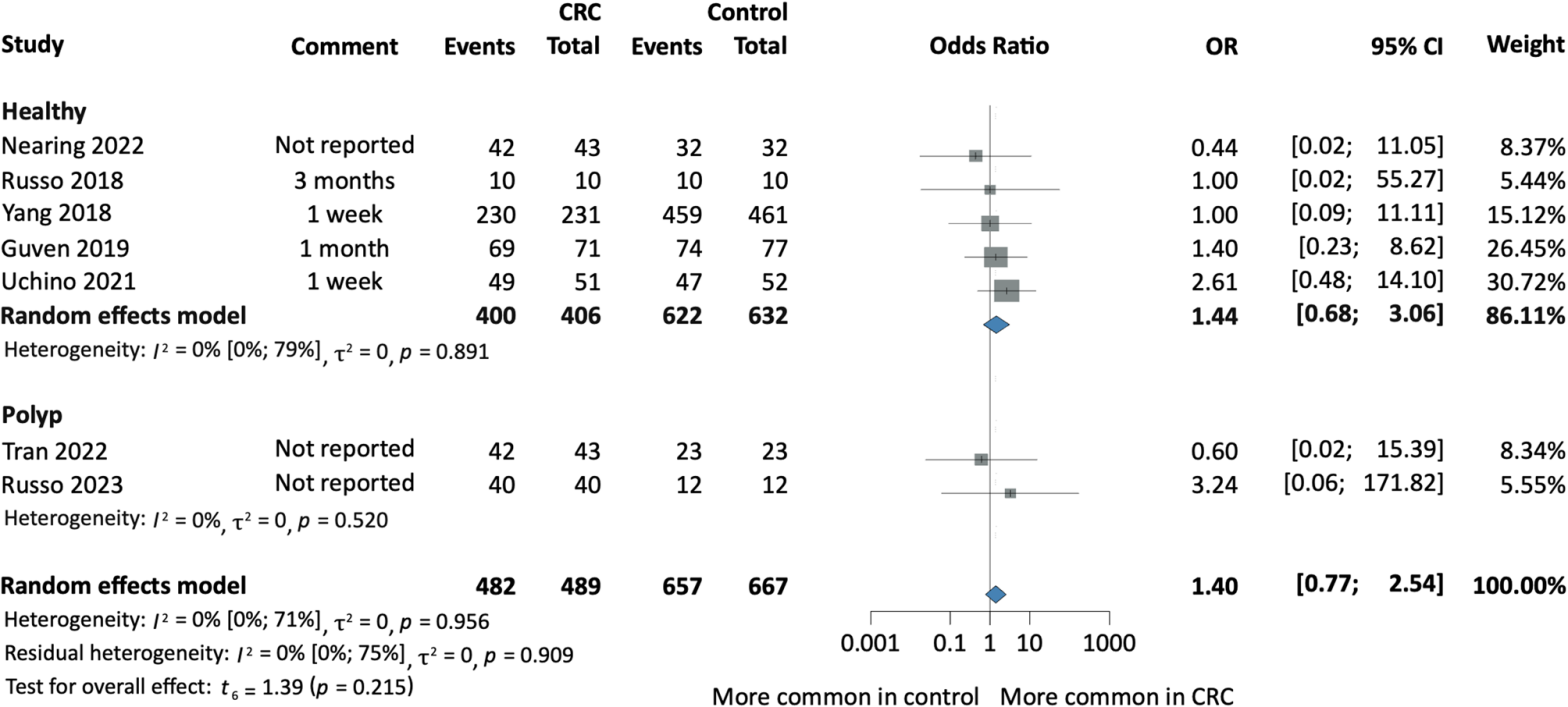
Forest plot representing the presence of salivary Fn among patients with CRC and Healthy individuals or colorectal polyps. *CI* confidence interval, *CRC* colorectal cancer, *OR* odds ratio.

**Fig. 3:**
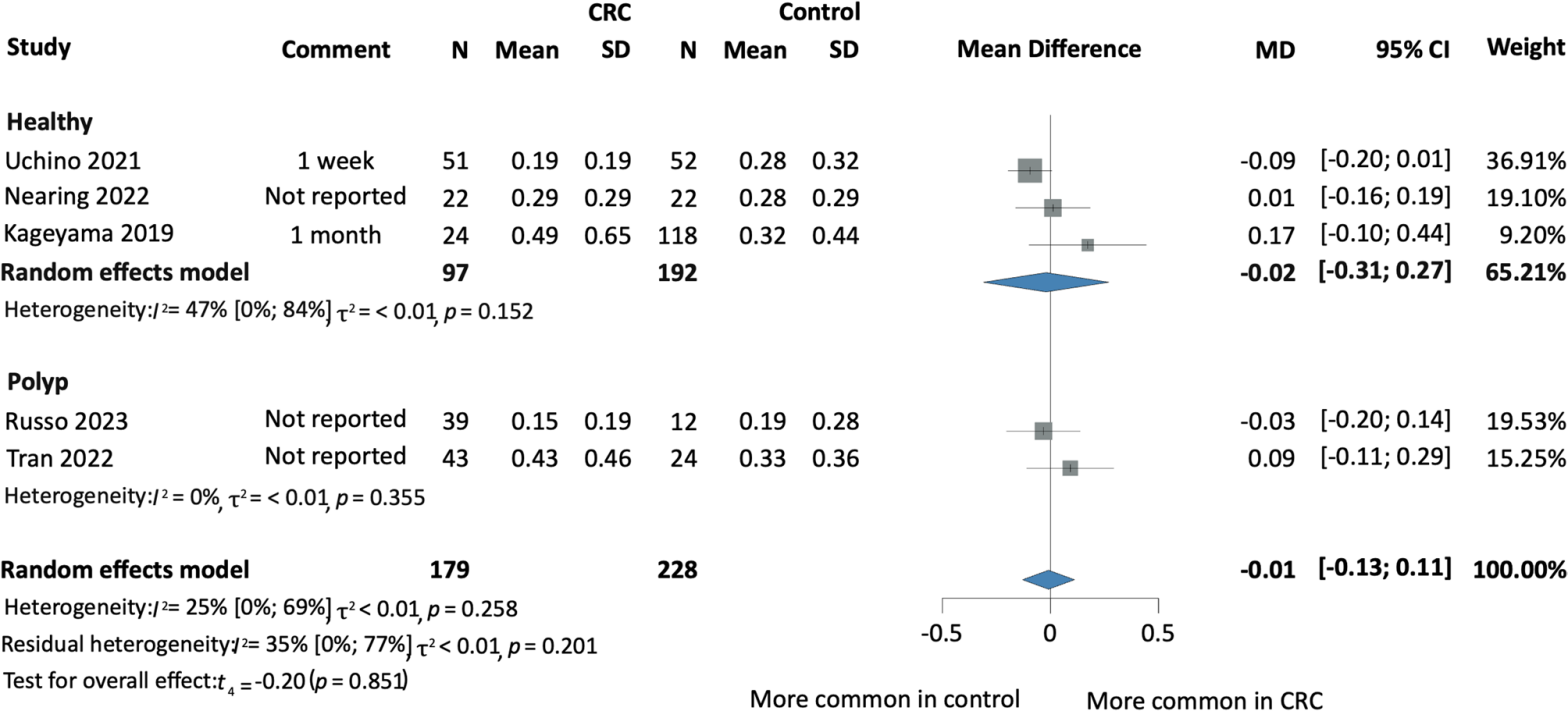
Forest plot indicating the presence of salivary Fn among CRC and Healthy individuals based on the antibiotic exclusion period. *CRC* colorectal cancer, *MD* mean difference, *CI* confidence interval.

**Fig. 4:**
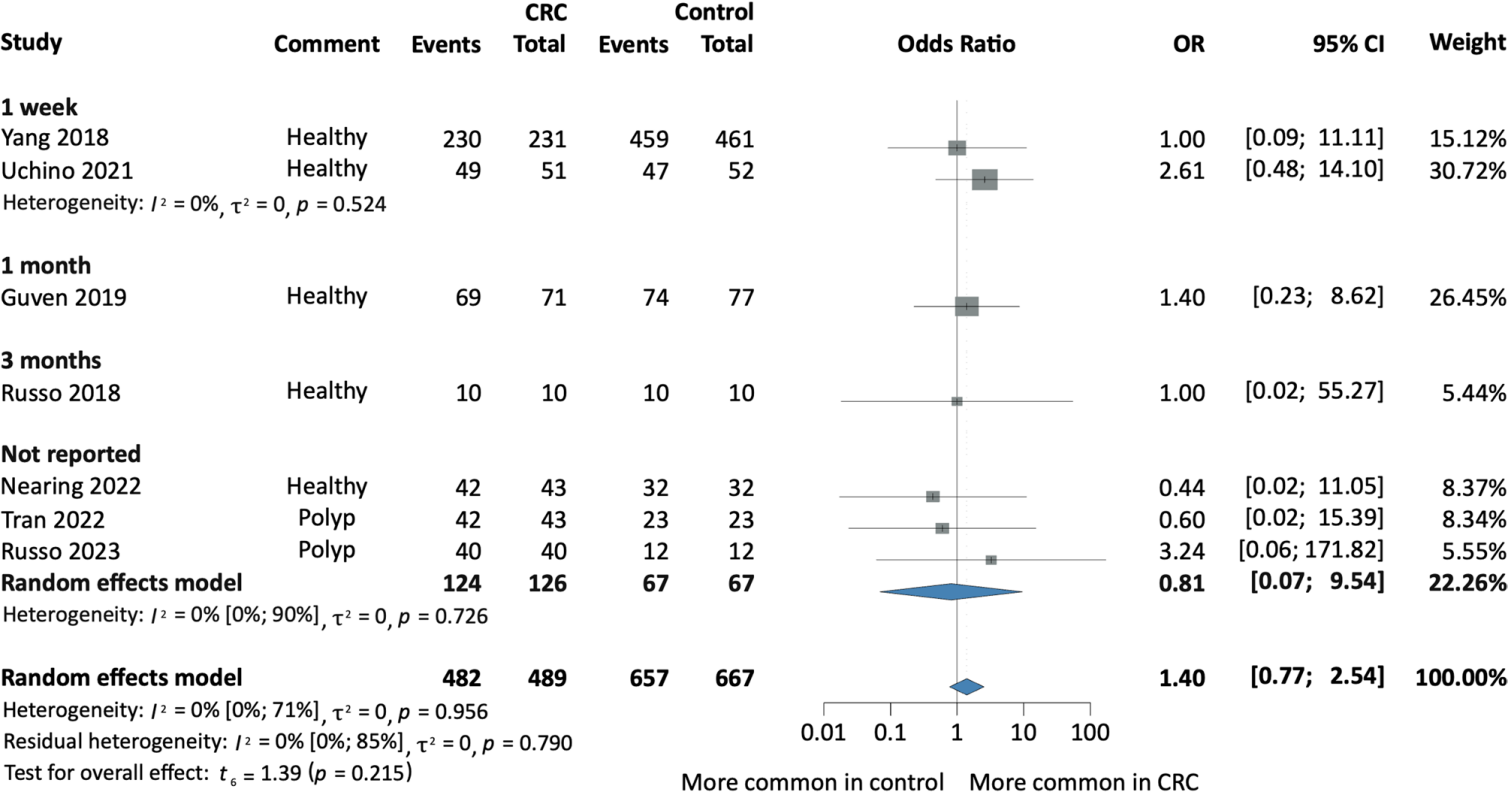
Forest plot indicating the presence of salivary Fn among CRC and Healthy individuals based on the antibiotic therapy prior to sample collection. *CRC* colorectal cancer, *OR* odds ratio, *CI* confidence interval.

**Fig. 5:**
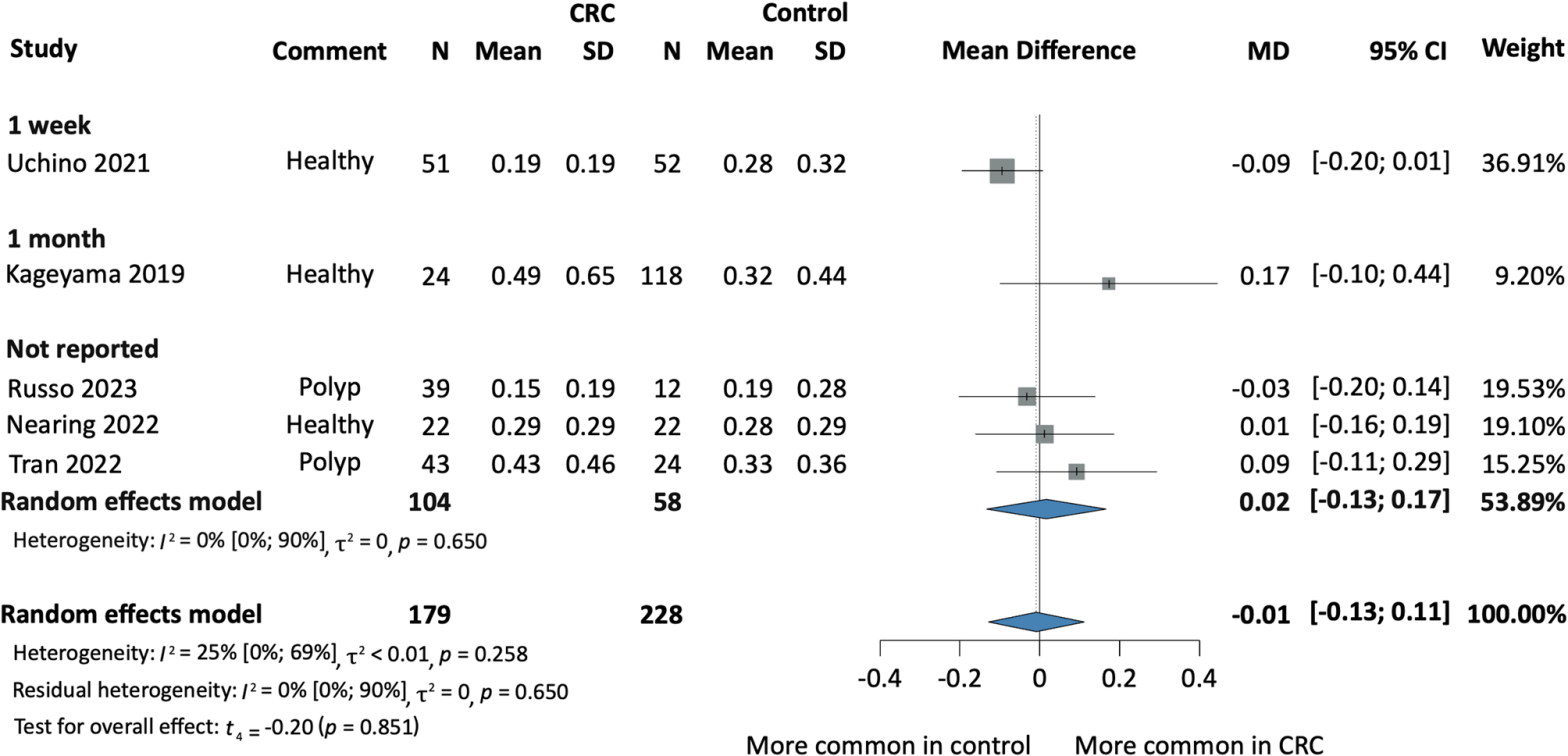
Forest plot indicating the relative abundance of salivary Fn among CRC and Healthy individuals based on the antibiotic therapy prior to sample collection. *CRC* colorectal cancer, *MD* mean difference, *CI* confidence interval.

#### No difference in the presence of salivary Fn

We found no statistically significant difference in the presence of salivary Fn among CRC patients compared to healthy controls (OR 1.44; 95% CI [0.68; 3.06]; *I*^2^ = 0% [0; 79%], *p* = 0.215). Similarly, we found no significant association in the presence of salivary Fn in CRC compared to the combined group of healthy and CRP controls (OR 1.40; 95% CI [0.77; 2.54]; *I*^2^ = 0% [0; 71%], *p* = 0.215) (Fig. 2).

#### No difference in the relative abundance of salivary Fn

We evaluated five prospective cohort and case-control studies to assess the relative abundance of Fn in the saliva of CRC patients compared to control groups^18–20,23,24^. The analysis indicated no statistically significant difference between CRC patients and healthy controls (MD -0.02; 95% CI [-0.31; 0.27]; *I*^2^ = 47% [0; 84%]). Similarly, there was no statistically significant difference between CRC and combined healthy/CRP controls (MD -0.01; 95% CI [-0.13; 0.11]; *I*^2^ = 25% [0; 69%], *p* = 0.851) (Fig. 3).

#### Subgroup analysis based on the duration of antibiotic therapy prior to sample collection

We conducted a subgroup meta-analysis based on the exclusion criteria according to antibiotic use within one week, one/three months prior to the sample collection. We found significant difference neither in the presence (OR 1.40; 95% CI [0.77; 2.54]; *I*^*2*^ *=* 0% [0; 71%], *p* = 0.215) nor in the relative abundance (MD -0.01; 95% CI [-0.13; 0.11]; *I*^*2*^ = 25% [0; 69%], *p* = 0.851) of salivary Fn between CRC and controls (CRP and healthy individuals) (Figs. 4 and 5).

#### Studies contained individual patient data (IPD)

We assessed the relative abundance of salivary Fn using density plots to examine its relationship with tumor location (left vs. right side), sex, age, and stage of cancer (I-IV). In all plots, the peak in density was in the lower range of salivary Fn relative abundance; however, we could not evaluate the higher relative abundance range of salivary Fn, as salivary Fn is rarely found in this range (Appendices 2–6).

#### Studies assessed qualitatively showed varied results

Four studies showed a higher relative abundance of salivary Fn in CRC, CRP, or a combined CRC/CRP than in healthy patients^2,17,21,26^; three found no significant association^22,27,29^. However, the quantitative analysis could not evaluate these studies due to the following limitations: (1) different reference genes^2,17^(2) lack of data^22,26,27,29^or (3) incomparable units^21^.

Zhang X et al. detected a higher salivary Fn median relative abundance in CRC than in colorectal adenoma (CRA, a subgroup of CRP), hyperplastic polyp (HP, a subtype of serrated CRP, which is a broader subgroup of CRP), and healthy controls (CRC 1.324 [IQR 0.338–5.134] vs. CRA 0.209 [IQR 0.147–0.688] vs. HP 0.076 [IQR 0.046–0.389] vs. healthy 0.105 [IQR 0.018–0.203]; *p* < 0.001, in their training cohort, and CRC 2.352 [IQR 1.290–6.743] vs. CRA 0.230 [IQR 0.125–1.310] vs. HP 0.190 [IQR 0.145–0.605] vs. healthy 0.250 [IQR 0.165–0.560]; *p* < 0.001) in their test cohort. These values were calculated as the ratio of Fn DNA (NusG gene) levels to the geometric mean of GAPDH and TERT gene levels^2^.

Janati et al. showed a higher salivary Fn median relative quantification in a combined group of CRC/CRP (0.345 [IQR 0.15–0.82]) than in healthy controls (0.12 [IQR 0.05–0.65]), measured as 2^−ΔCq^ using Fn 16 S rRNA as the target gene and MEFE as a reference gene^17^.

Zhang L et al. found an increased abundance of salivary Fn in CRP patients than in healthy controls (*P* < 0.05) using the total bacterial 16 S rRNA full-length^26^.

Wang et al. detected no significant difference in the relative abundance of salivary Fn in CRC (9.91 [IQR 8.799–11.216]) than in healthy controls (10.125 (IQR 9.1584–11.4306); *p* = 0.527) measured as the difference between total bacteria 16 S rRNA Cq and Fn 16 S rRNA Cq values. Lower Cq values mean a higher relative abundance of the gene^25^.

Similarly, neither Yang nor Russo found an association between salivary Fn and CRC using the 16 S rRNA target gene^23,27^. Finally, Guven et al. evaluated the absolute quantity of salivary Fn and found higher mean levels in CRC patients (6.89 ± 1.07 log10 copies/ml) than in healthy controls (6.35 ± 0.78 log10 copies/ml).(*p* = 0.001)^21^.

#### Diagnostic value of salivary Fn in the detection of CRC

Only two studies assessed the diagnostic accuracy of salivary Fn in detecting CRC; thus, we could not conduct a quantitive analysis. Zhang X et al. reported that salivary Fn can detect CRC with high diagnostic accuracy (area under the curve (AUC) = 0.841; 95% CI 0.797–0.879), achieving a sensitivity of 71.5% and a specificity of 82.1% in the training set^2^. Similarly, high diagnostic accuracy was achieved in the test cohort (AUC = 0.860; 95% CI 0.774–0.922), reaching a sensitivity of 86.7% and a specificity of 67.2% ^2^. Guven et al. achieved no significant results when assessing the diagnostic accuracy of salivary Fn for CRC diagnosis^21^.

### Risk of bias assessment

**Fig. 6:**
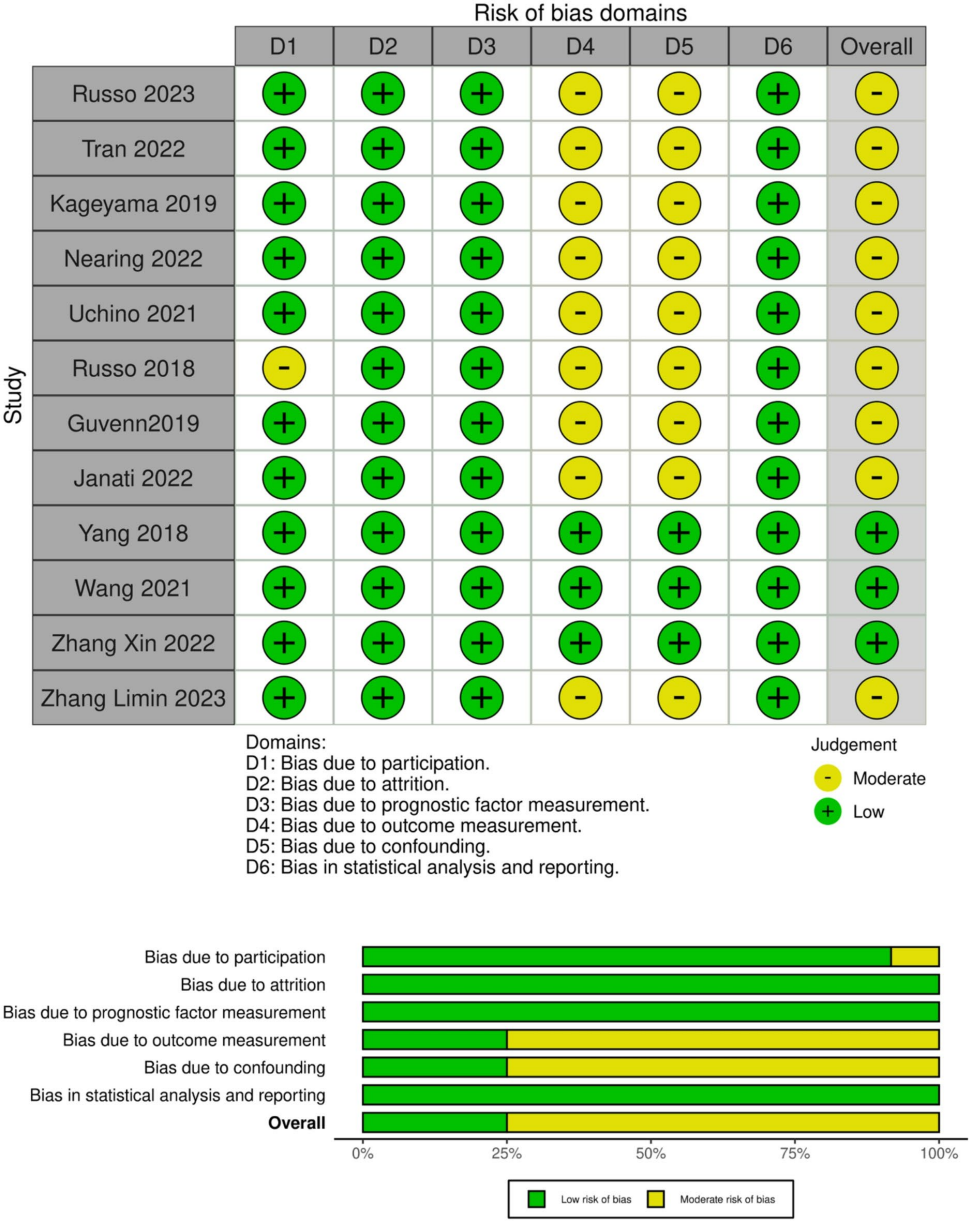
Risk of bias assessment using the QUIPS tool across studies with color codes representing levels of bias: green for low risk and yellow for moderate risk.

The risk of bias was evaluated using the QUIPS tool^30^. One study demonstrated a potential bias in the study participation domain due to a small sample size^22^. Three studies showed a low overall risk of bias^2,25,27^. The other nine studies presented a moderate overall risk of bias, as they did not mention whether blinding was used, demonstrating a potential bias in the outcome measurement domain (Fig. 6). Additionally, they did not adjust the confounding factors, showing a possible bias in the study confounding domain^17–24^.

## Discussion

The major findings of this research revealed no significant difference in the relative abundance of salivary Fn in patients with CRC, compared to CRP, and healthy controls. Not surprisingly, we found no significant difference in the presence of salivary Fn in CRC patients compared to CRP and healthy controls, as Fn is part of the oral microbiome. The diagnostic accuracy of salivary Fn in detecting CRC could not be assessed by the meta-analysis due to the low number of eligible studies.

Previous meta-analyses have investigated the association between CRC and Fn using fecal samples or tissue biopsies. Villar-Ortega et al. showed significantly higher odds for the positivity of Fn in CRC than in CRA tissue biopsies (OR 3.244; 95% CI: 2.359 − 4.462), as well as in healthy controls (OR 4.558; 95% CI: 3.312–6.272)^31^. Kim et al. indicated that high Fn levels in tissue biopsies of CRC patients are associated with poor overall survival (OS) (Hazard ratio (HR) 1.58; 95% CI 1.28–1.94), disease-free survival (HR 1.76; 95% CI 1.06–2.93), and cancer-specific survival (HR 1.72; 95% CI 1.05–2.83)^32^. Huangfu et al. found a similar association with OS (HR 1.40; 95% CI: 1.40–1.63)^14^. Zhang X et al. assessed the diagnostic accuracy of fecal Fn. They found a promising diagnostic accuracy for CRC with a sensitivity of 71% (95% CI 61%-79%) and a specificity of 76% (95% CI 66%‐84%), but a much lower value for the diagnosis of CRA, with a sensitivity of 36% (95% CI 27%‐46%) and a specificity of 73% (95% CI 65%‐79%)^33^. In light of these meta-analyses and the fact that Fn may originate from the oral cavity and colonize the CRC tissues^11^we expected to detect similar outcomes using saliva samples.

The results of our study may be attributed to the fact that various factors, including age, gender, diet, and oral hygiene, influence saliva composition, and its microbiome. Consequently, it exhibits significant individual variations^34^. This presents substantial challenges in acquiring standardized saliva samples, diminishing the comparability and reliability of the outcomes^35^.

In contrast to the results of this research, two of the included studies showed an association between salivary Fn relative abundance and CRC^2,21^. One of those studies did not assess essential cofounders, such as periodontal disease, which is associated with Fn. Furthermore, the control group was not evaluated for precancerous lesions using colonoscopy, potentially confounding the bacterial analysis^21^.

This meta-analysis could not assess the diagnostic accuracy of salivary Fn in detecting CRC. Nonetheless, it is essential to acknowledge the inherent advantages of saliva as a diagnostic medium. Notably, detecting alterations in saliva composition may still serve as a valuable diagnostic tool in other contexts. A significant advantage of using saliva as a diagnostic tool is its non-invasive nature. Unlike blood or tissue biopsies, saliva collection is easy and painless, which improves patient compliance and facilitates repeated sampling over time^36^. In addition, saliva collection poses less risk to patients and healthcare providers than other sample types. It has a lower risk of exposure to blood-borne pathogens such as HIV or hepatitis viruses, and unlike blood, it does not clot and is, therefore, easier to manipulate^37^. Saliva testing is generally more cost-effective compared to fecal tests. It requires less specialized equipment and training for collection and processing, making it a more economical option for large-scale screenings and routine diagnostics^38^.

We could assess the distribution of salivary Fn among different subgroups using density plots (appendices 2–6). Four studies indicated that CRC and CRP patients are more likely to have a low range of salivary Fn relative abundance than healthy controls^18,23,24,28^. From two studies, it was observed that patients with right-sided tumors are more likely to have a low range of salivary Fn relative abundance than those with left-sided tumors^23,24^. Interestingly, when analyzing the salivary Fn relative abundance based on sex, we found that males with CRC are more likely to have salivary Fn relative abundance at the low range than women; however, females and males with CRP have the same likelihood of having salivary Fn relative abundance in the low range^18,23,24^. Assessing the salivary Fn relative abundance among CRC patients at different stages, it was noted that stage I CRC patients are more likely to have Fn relative abundance at the low range than the other stages^23,24^. Tran et al. analyzed the salivary Fn relative abundance among CRP and CRC at different age groups. However, no correlation between the salivary Fn relative abundance and the patient’s age could be noticed^24^. Looking at the higher ranges of salivary Fn, it was impossible to assess the relative abundance of the bacteria at these ranges. One possible explanation is that after a certain threshold, Fn can not be detected in saliva.

Abed et al. observed that Fn may be transmitted from the oral cavity to the bloodstream^11^. This hematogenous spread allows Fn to reach distant organs, including the pancreas, promoting cancer cell proliferation and migration^8^. Similar to pancreatic cancer, Fn can translocate from the oral cavity to breast tissue through multiple routes, including the mammary-intestinal axis, direct nipple contact, and hematogenous transmission^39^. In addition, Parhi et al. have indicated that Fn colonizes breast cancer and is secondary to tumor initiation^40^.

Even though Fn is primarily known for its association with various diseases, particularly CRC and periodontal disease, it is essential to note some of its positive effects. The bridging role of Fn is crucial for developing a stable and diverse oral microbiome, which is vital for maintaining oral health. A balanced oral microbiome can help prevent the overgrowth of pathogenic bacteria and maintain oral homeostasis^7^. Hsieh et al. have shown the ability of Fn to induce an interferon response in gastric cancer cells, which might affect immune system activation and regulation. Although this immune modulation can contribute to disease progression in some contexts, it may also have potential therapeutic implications in modulating immune responses^41^.

While Fn plays a significant role in CRC development, other members of the oral microbiota may also be associated with CRC. Guven et al. found increased levels of Streptococcus gallolyticus in the saliva of CRC patients^21^. Kageyama et al. indicated a higher relative abundance of other bacteria, such as Porphyromonas gingivalis, Streptococcus parasanguinis, Neisseria species, and Actinomyces odontolyticus, in CRC patients. To exclude the influence of periodontal infection on the oral microbiota, they conducted a dental examination and found no significant difference in the periodontal health of CRC patients and healthy controls; however, the small sample size may limit this finding^19^. Furthermore, Kudra et al. indicated that *P. gingivalis* increases the risk for CRC and perhaps even stimulates its growth. Several studies imply that *P. gingivalis* would be beneficial as a non-invasive biomarker in the future. However, since these bacteria induces periodontal disease, which occurs much more frequently than CRC, it may be of little value. Moreover, establishing microbial biomarkers is a complex and challenging task. Various factors must be taken into consideration, including the type of samples (dental plaque, stimulated or unstimulated saliva), analytical methods, CRC stage, age, dental health, and oral hygiene levels^42^.

The limitations of individual studies should be considered; Zhang. X et al. could not follow up with patients long enough^2^. Kageyama et al. indicated that the sample size was insufficient to confirm the association independently of the gingival condition^19^. Guven et al. used the qPCR method rather than NGS. In addition, potential confounders were not assessed^21^. Furthermore, the observational design of the studies could not explain the causal relationship between salivary Fn and CRC. Chen et al. explained how the structure of a study should be performed to prove causality^43^. Thus, it is important to note that studies done so far included small sample sizes making difficult to control for the various confounding factors when assessing the association between salivary Fn and CRC.

The limitations of this research were: (1) the use of different DNA extraction kits among studies, which may affect microbiota profiling^44^. Variations in DNA extraction methodologies and sequence curation steps employed among laboratories - the processes of filtering and processing raw genetic or microbial sequence data - significantly reduce consistency and comparability between studies, potentially leading to confounding results^45^; (2) the expression of salivary Fn levels was inconsistent among studies, which prevented us from conducting a quantitative assessment in some cases; (3) there was a low number of eligible studies, and about half of the included studies could not be analyzed by the meta-analysis; (4) the small number of studies did not allow us to interpret the diagnostic accuracy of salivary Fn quantitively.

On the other hand, the strengths of this study should also be mentioned. We adhered to the pre-registered protocol and applied a strict methodology. Where possible, data on possible influencing factors, such as patient sex, age, tumor stage, and location, were extracted from each patient’s data. Lastly, studies from different regions with low to moderate risk of bias were included.

Implementing scientific findings in practice is crucial^46,47^. Therefore, on the basis of the major findings of this research, salivary Fn alone should not be recommended as a biomarker for CRC. Adopting unified methods for detecting salivary Fn is essential to enhance the reliability of the results. Moreover, we encourage researchers to investigate other non-invasive biomarkers that can replace colonoscopy.

## Conclusion

The findings of this research show that the presence and relative abundance of salivary Fn are not associated with CRC. Therefore, salivary Fn may not be a reliable biomarker for detecting CRC.

## Methods

This systematic review and meta-analysis was conducted using the PRISMA 2020 guidelines^48^ and followed the Cochrane Handbook^49^. The study protocol was registered on PROSPERO (registration number CRD42023474939), and we adhered to it.

### Eligibility criteria

The PECO and PIRD frameworks were used to address the clinical questions. We included studies if they reported on salivary samples of adults over 18 years old (P) with colorectal cancer (E), polyps (C), or healthy individuals (C). Moreover, articles had to contain data on the presence, quantity, or relative abundance of salivary Fn (O). In addition, we collected data on the diagnostic performance of salivary Fn if they reported on adults over 18 years old (P), salivary Fn (I), histology (R), and CRC (D). Interventional and analytical observational studies were eligible. Reports were excluded if the investigators obtained samples from crevicular fluid or subgingival plaque. Also excluded were studies that contained other gastrointestinal diseases.

### Information source

The systematic search was conducted on the MEDLINE (via Pubmed), Embase, Cochrane Central Register of Controlled Trials (CENTRAL), and Scopus databases on November 25, 2023.

### Search strategy

The systematic search was performed using a search key consisting of four domains: (1) saliva AND (2) fusobacterium AND (3) colorectal AND (4) cancer. See Appendix 1 for a detailed search key. No restrictions or filtering options were applied.

### Selection process

Duplicate articles were automatically and manually removed using the citation manager Endnote 20. Two independent review authors (EG and BZ-S) used the Rayyan Intelligent Systematic Review program^50^ to select articles by title, abstract, and full texts based on eligibility criteria. A third author (PM) then resolved the conflicts.

### Data collection process and data items

Two independent authors (EG and BZ-S) extracted data from included studies based on eligibility criteria. The following data were extracted into a Microsoft Excel spreadsheet (Microsoft 2016, Redmond, WA, USA): first author, publication year, study period, sample size, and population characteristics such as age and sex distribution. We also collected information about the presence/abcsence, quantity, and relative abundance of salivary Fn, the bacterial analysis methods, and the DNA extraction kit type. The relative abundance of salivary Fn was manually extracted from the SRA run selector archive by National Library of Medicine in four studies^18,20,23,24^whereas, in one study, it was provided by the author^19^ .

### Study risk of bias assessment

Two independent authors (EG and BZ-S) assessed the risk of bias using the Quality In Prognosis Studies (QUIPS) tool^30^. This tool evaluates six domains: study participation, study attrition, prognostic factor measurement, outcome measurement, study confounding, and statistical analysis and reporting.

### Statistical methods

As considerable between-study heterogeneity was assumed in all cases, a random-effects model was used to pool effect sizes. Statistical analysis was performed with R 4.3.2^51^. and package meta 6.5.0^52^.

CRC patients were compared to healthy, or healthy and CRP patients by calculating the mean differences with 95% confidence intervals of Fn relative abundances. Sample sizes, mean and standard deviation values were extracted from the studies to calculate mean differences. Mean differences were calculated by extracting the mean values of the control group from the mean values of the CRC group.

For the presence of salivary Fn, odds ratios with 95% confidence intervals were used as outcome measures. Results are presented as the odds of the CRC group compared to the odds of the same event in the control group. Results were considered statistically significant if the pooled CI did not contain the null effect value. Egger’s test and funnel plots were planned to visualize publication bias if at least ten studies were involved in the analysis. Pooled OR was calculated using the Mantel-Haenszel method^53,54^. Confidence intervals were created with the Paule-Mandel method^55^as recommended by Veroniki et al.^56^.

Pooled mean differences were computed with the inverse variance method. The restricted maximum-likelihood estimator was used with the Q profile method for confidence intervals by Harrer et al.^57^and Veroniki et al.^56^. Hartung-Knapp adjustments were also applied^58,59^ We summarized the results on forest plots. Between-study heterogeneity was assessed with the Higgins and Thompson’s I² statistic^60^and the Cochrane Q test Harrer et al.^57^.

## Electronic supplementary material

Below is the link to the electronic supplementary material.

Supplementary Material 1

## Data Availability

The datasets used and/or analysed during the current study are available from the corresponding author on reasonable request.
