# Supplementary material for: The presence and relative abundance of salivary Fusobacterium nucleatum are not associated with colorectal cancer: a systematic review and meta-analysis
Source: Sci Rep. 2025 Jul 10;15:24815. doi: 10.1038/s41598-025-07465-w (PMC12246035; doi:10.1038/s41598-025-07465-w)
Supplement: Supplementary file 1 — Supplementary Material 1 [file 41598_2025_7465_MOESM1_ESM.docx]

**Supplementary Material**

**The presence and relative abundance of salivary**

**Fusobacterium nucleatum are not associated with**

**colorectal cancer: a systematic review and**

**meta-analysis**

Ellay Gutmacher^1^, Bálint Zsombor Sárai^1,2^, Petrana Martineková^1^, Szilvia Kiss-Dala^1,5^, Gergely Agócs^1,4^, Péter Hegyi^1,4,5^, Andrea Bródy*^1,6^, Ákos Zsembery*^1,7^

*These authors had an equal contribution to the manuscript, and both share the last authorships

1. Centre for Translational Medicine, Semmelweis University, Budapest, Hungary
2. Department of Public Dental Health, Semmelweis University, Budapest, Hungary
3. Institute of Biophysics and Radiation Biology, Semmelweis University, Budapest, Hungary
4. Institute of Pancreatic Diseases, Semmelweis University, Budapest, Hungary
5. Institute for Translational Medicine, Medical School, University of Pécs, Pécs, Hungary
6. Department of Oral Diagnostics, Semmelweis University, Budapest, Hungary
7. Department of Oral Biology, Faculty of Dentistry, Semmelweis University, Budapest, Hungary

**Corresponding author**

Ákos Zsembery MD, PhD

Postal address: H-1089 Budapest, Nagyvárad tér 4, Hungary

Tel.: +(36-20) 4633766

E-mail address: [zsembery.akos](mailto:zsembery.akos)@semmelweis.hu

**Appendix 1-** Search key used for the systematic search on 25.11.2023

**PUBMED**: 2,629

(spitt* OR saliv* OR "oral" OR "mouth" OR "sputum") AND (fusobact* OR microb*) AND ("colorectal" OR "colon" OR "colonic" OR "rectum" OR "rectal" OR "gut" OR "intestinum" OR "intestine" OR "intestines" OR "intestinal") AND (cancer* OR "neoplasia" OR "neoplasms" OR "neoplasm" OR "malignant" OR "malignancy" OR "malignancies" OR tumor* OR tumour* OR polyp* OR precarcinoma* OR precanc* OR adenoma* OR "adenomatous")

**EMBASE**: 7,772 (Advanced search)

(spitt* OR saliv* OR mouth OR oral OR sputum) AND (fusobact* OR microb*) AND (colorectal OR colon OR colonic OR intestinum OR intestine OR intestines OR intestinal OR rectum OR rectal OR gut) AND (cancer* OR neoplasia OR neoplasm OR neoplasms OR malignant OR malignancy OR malignancies OR tumor* OR tumorous OR polyp* OR precarcinoma* OR precanc* OR adenoma* OR adenomatous)

**CENTRAL**: 416 (All text)

(spitt* OR saliv* OR mouth OR oral OR sputum) AND (fusobact* OR microb*) AND (colorectal OR colon OR colonic OR intestinum OR intestine OR intestines OR intestinal OR rectum OR rectal OR gut) AND (cancer* OR neoplasia OR neoplasm OR neoplasms OR malignant OR malignancy OR malignancies OR tumor* OR tumorous OR polyp* OR precarcinoma* OR precanc* OR adenoma* OR adenomatous)

**SCOPUS**: 3,383 (Abstract, Title, Keywords)

( spitt* OR saliv* OR mouth OR oral OR sputum ) AND ( fusobact* OR microb* ) AND (colorectal OR colon OR colonic OR intestinum OR intestine OR intestines OR intestinal OR rectum OR rectal OR gut ) AND ( cancer* OR neoplasia OR neoplasm OR neoplasms OR malignant OR malignancy OR malignancies OR tumor* OR polyp* OR precarcinoma* OR precanc* OR adenoma* OR adenomatous )

**Appendix 2**- Density plot representing the relative abundance of salivary Fn among CRC, CRP, and healthy controls (^1-4^).


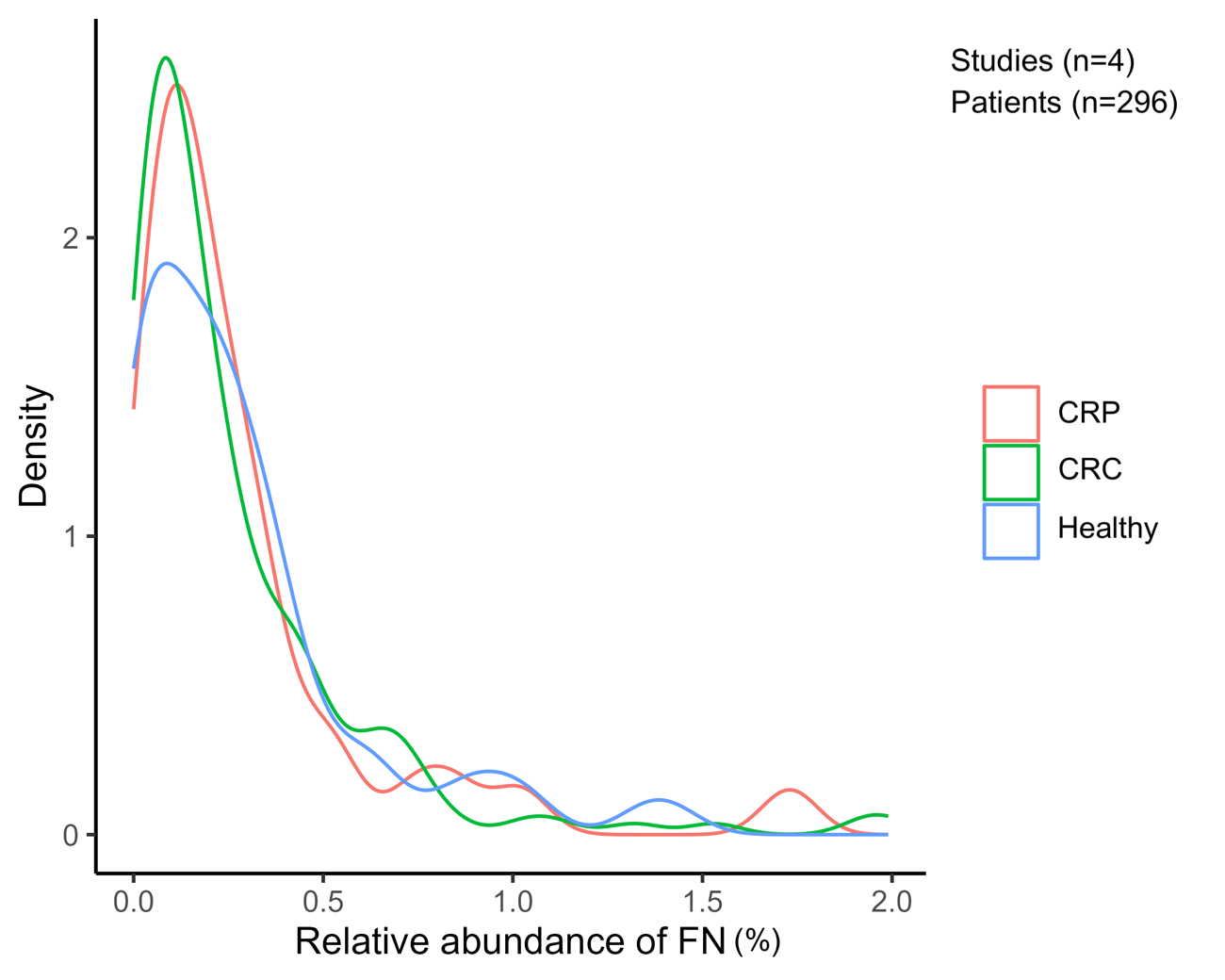


CRP - Colorectal polyp: CRC- Colorectal cancer, n – Number.

**Appendix 3**- Density plot representing the relative abundance of salivary Fn based on the tumor’s location among CRC and CRP patients (^3,4^).


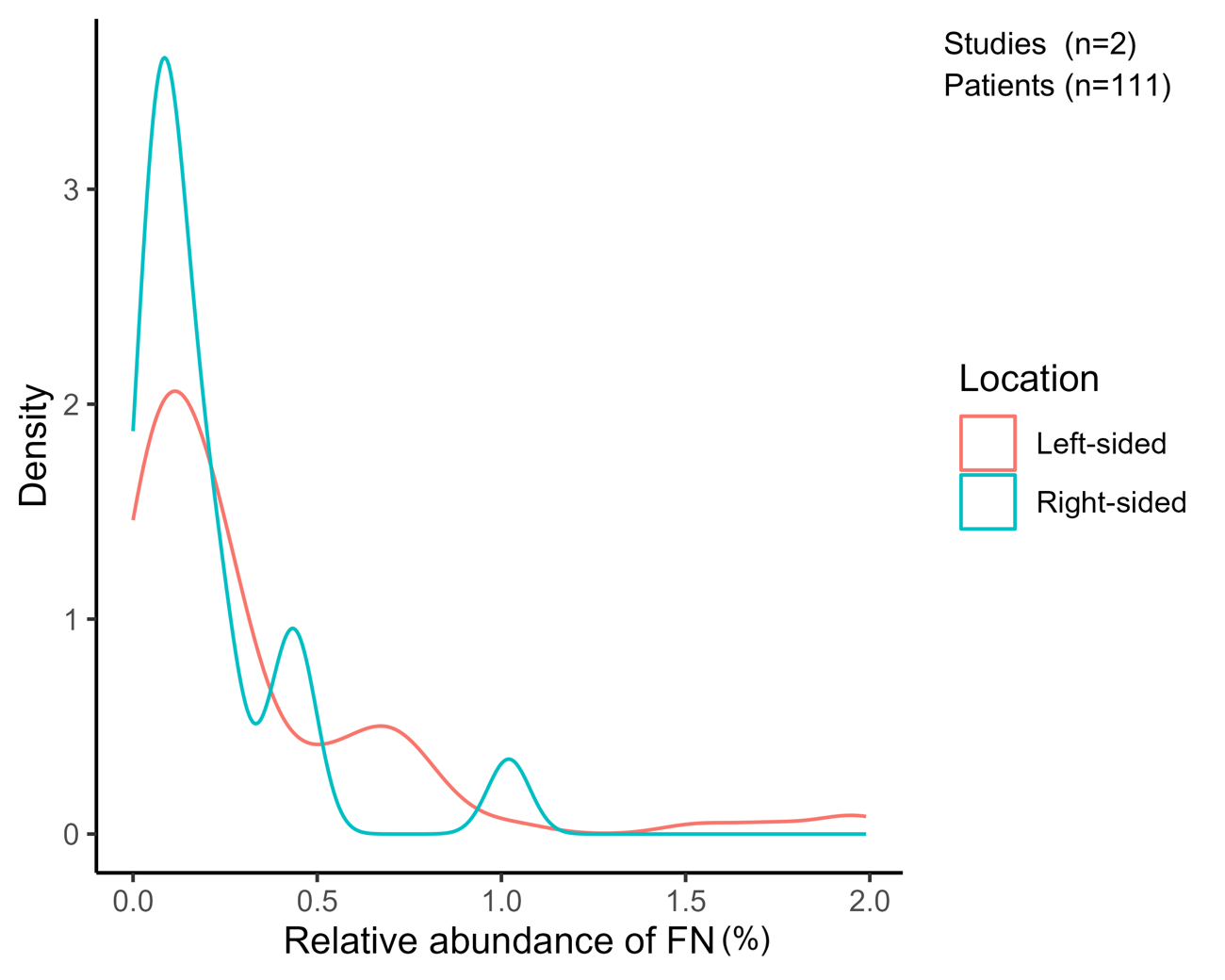


**Appendix 4** - Density plot representing the relative abundance of salivary Fn among Females and Males with CRP and CRC, or healthy (^2-4^).


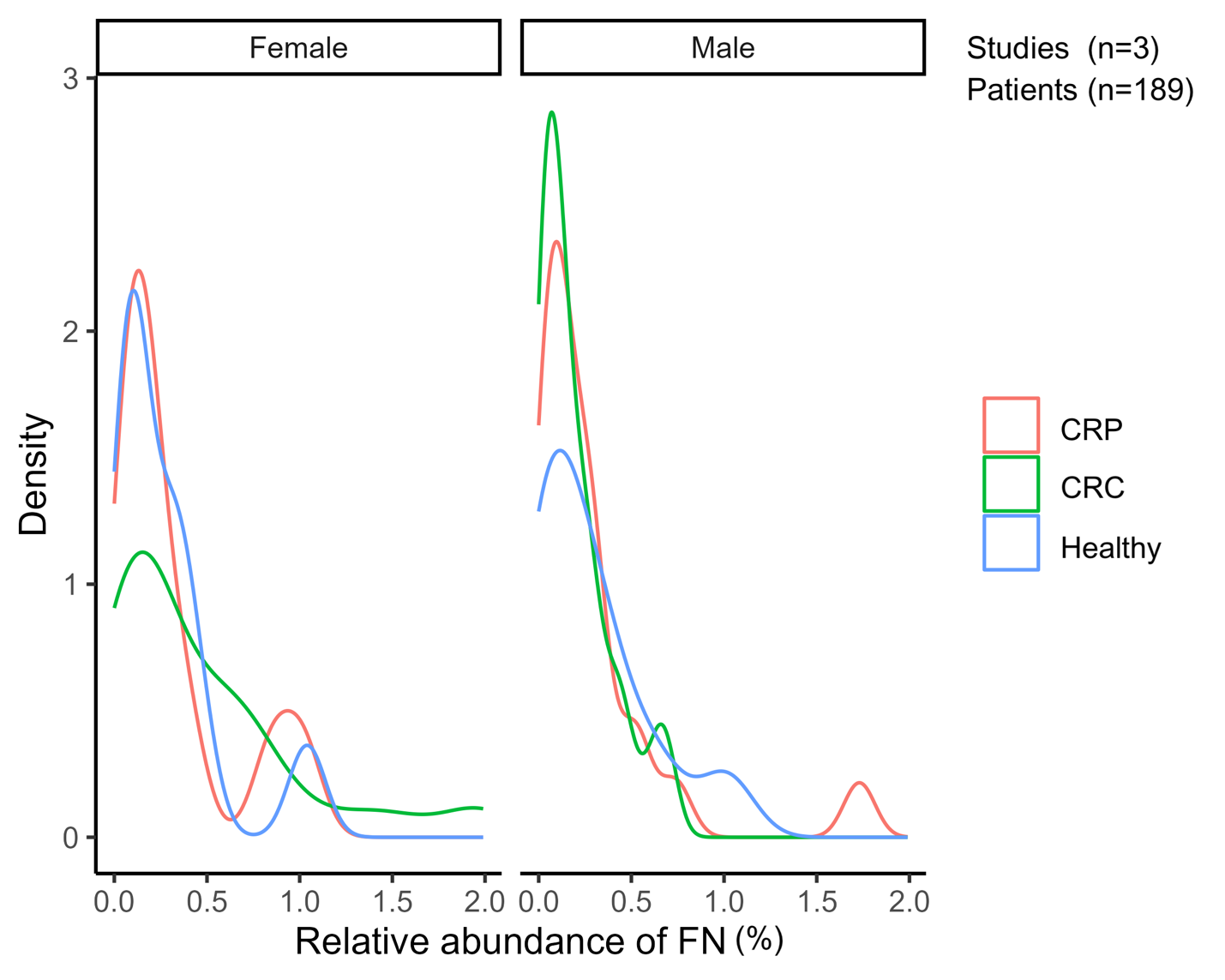


**Appendix 5** - Density plot representing the relative abundance of salivary Fn among CRC patients at different stages (^3,4^).


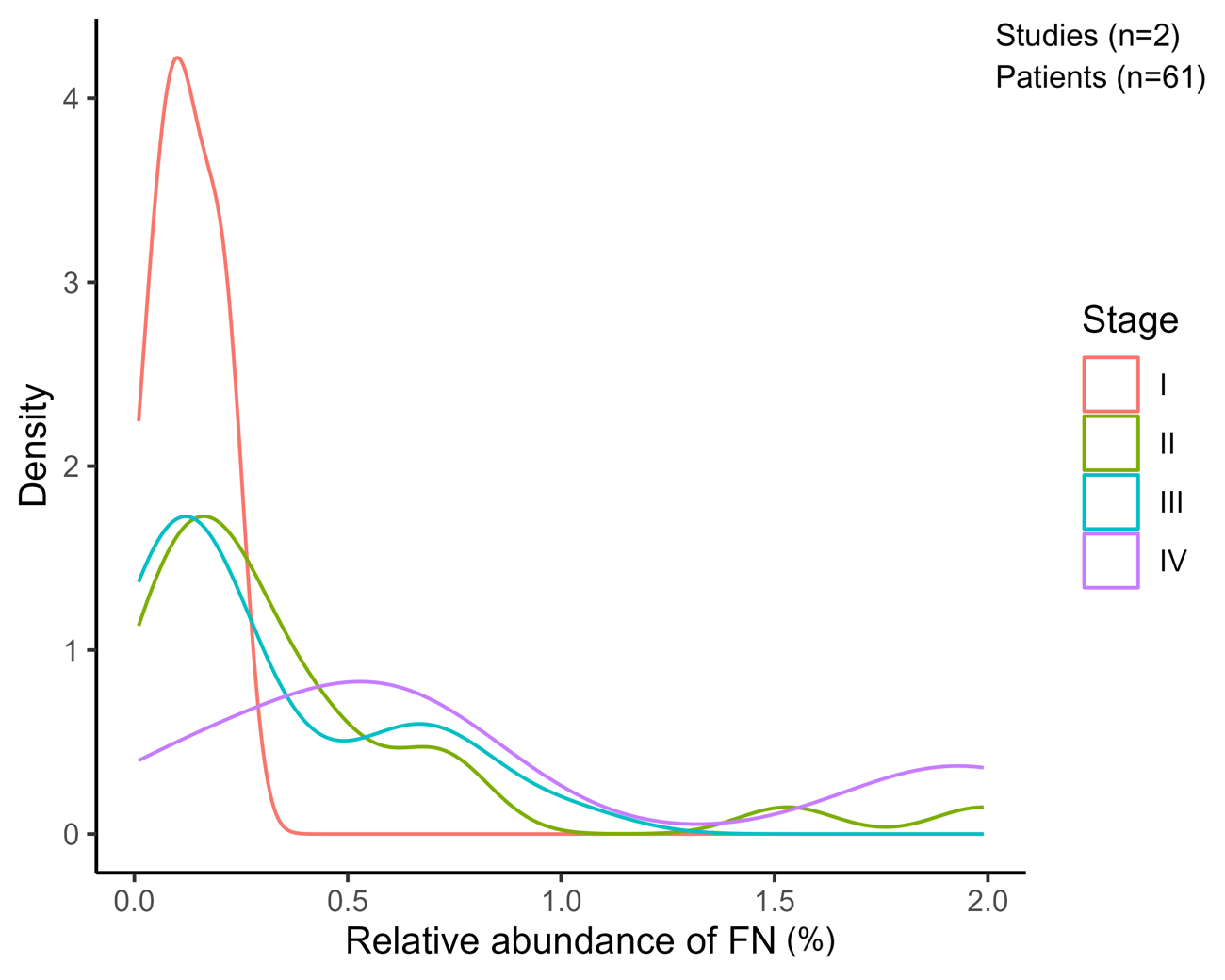


**Appendix 6** - Density plot representing the relative abundance of salivary Fn among CRP, and CRC patients at different stages (^4^).


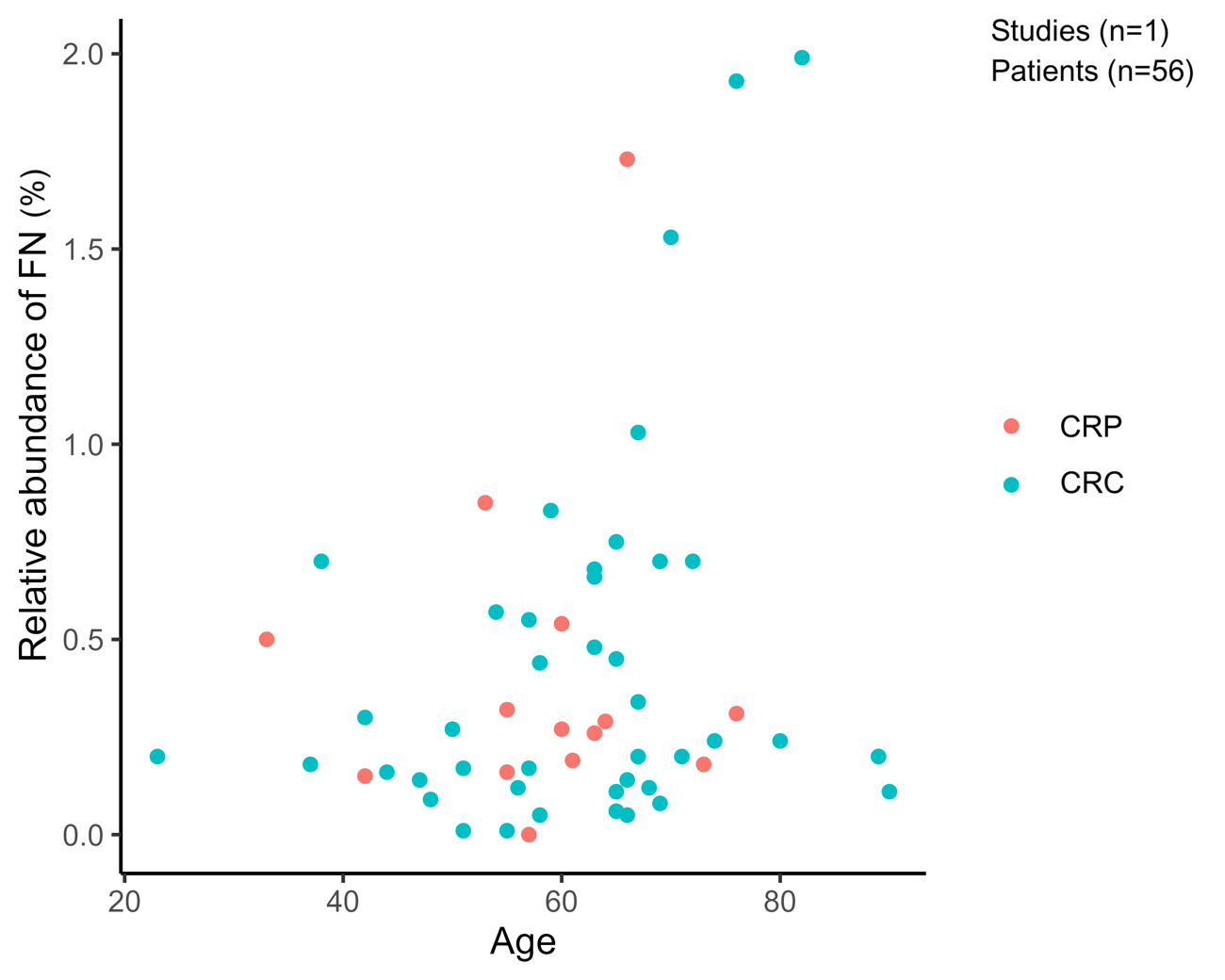


**Appendix 7 –** Table of excluded studies

| First Author | Title | DOI | Reason for exclusion |
| --- | --- | --- | --- |
| I. Kato | Oral microbiome and history of smoking and colorectal cancer | 10.5430/jer.v2n2p92 | Different population (Buccal cells mouth wash) |
| H. Morsi, | Detection of Fusobacterium nucleatum subspecies in the saliva of pre-colorectal cancer patients, using tandem mass spectrometry | 10.1016/j.archoralbio.2021.105337 | Different outcome (Fn proteins/strains) |
| Y. Shimomura | Strain-level detection of Fusobacterium nucleatum in colorectal cancer specimens by targeting the CRISPR-Cas region | 10.1128/spectrum.05123-22 | Different outcome (no levels of Fn) |
| Y. Komiya | Patients with colorectal cancer have identical strains of Fusobacterium nucleatum in their colorectal cancer and oral cavity | 10.1136/ gutjnl-2018-316661 | Different outcome (strains of Fn) |
| C. Zhang | Combined Non-Invasive Prediction and New Biomarkers of Oral and Fecal Microbiota in Patients With Gastric and Colorectal Cancer | 10.3389/fcimb.2022.830684 | Different population (oral swabs) |
| S. Zhang | Human oral microbiome dysbiosis as a novel non-invasive biomarker in detection of colorectal cancer | 10.7150/thno.49515 | Different population (oral swabs) |
| S. Segato | Is there any correlation between oral and intestinal fusobacterium nucleatum with colorectal adenoma and adenocarcinoma? | <https://doi.org/10.1016/S1590-8658(20)30835-5> | Different population (periodontal filter cones) |
| A. Scott | Direct isolation of oral-and tumour-derived fusobacterium species in patients with colorectal neoplasia | 10.1177/2050640616663689 | Different population (periodontal swabs) |
| X. Mai | Pathogenic oral bacteria and risk of incident cancer in postmenopausal women: The Buffalo OsteoPerio Study | 10.1158/1538-7445.AM2015-848 | Different population (subgingival plaque samples) |
| S. Rezasoltani | Oral Microbiota as Novel Biomarkers for Colorectal Cancer Screening | 10.3390/cancers15010192 | Different exposure (no information on salivary Fn) |
| T. Yoshihara | A prospective interventional trial on the effect of periodontal treatment on Fusobacterium nucleatum abundance in patients with colorectal tumours | 10.1038/s41598-021-03083-4 | No control group |
| C. Nardelli | Oral and colon microbiome in colorectal cancer patients with obesity: A pilot study | N/A | No full text |
| T. S. Schmidt | Extensive transmission of microbes along the gastrointestinal tract | 10.7554/eLife.42693 | Only sub-species of Fn |
| Y. Wang | Alterations in the oral and gut microbiome of colorectal cancer patients and association with host clinical factors | 10.1002/ijc.33596 | Different population (details only on fusobacterium genus) |
| H. N. H. Tran | Mucosal microbiomes and Fusobacterium genomics in Vietnamese colorectal cancer patients | 10.1101/2022.02.25.481918 | Overlapping population (duplicate of an included study) |
| J. T. Nearing | Investigating the oral microbiome in retrospective and prospective cases of prostate, colon, and breast cancer | 10.1038/s41522-023-00391-7 | Overlapping population (duplicate of an included study) |
| Y. Komiya | Association of Fusobacterium Nucleatum in Colorectal Cancer and Oral Cavity | 10.1016/S0016-5085(19)38612-3 | No full text |
| S. Rezasoltani | Oral Microbiota as Novel Biomarkers for Colorectal Cancer Screening | 10.3390/cancers15010192 | Overlapping population (duplicate of an excluded study) |
| P. Pignatelli | The Potential of Colonic Tumor Tissue Fusobacterium nucleatum to Predict Staging and Its Interplay with Oral Abundance in Colon Cancer Patients | 10.3390/cancers13051032 | No control group |
| Y. Komiya | Association of Fusobacterium Nucleatum in Colorectal Cancer and Oral Cavity | http://dx.doi.org/10.1016/S0016-5085(19)38612-3 | Overlapping population (duplicate of an excluded study) |
| B. Flemer | The oral microbiota in colorectal cancer is distinctive and predictive | 10.1136/gutjnl-2017-314814 | Different population (oral swabs) |
| S. Rezasoltani, | Oral Microbiota as Novel Biomarkers for Colorectal Cancer Screening | 10.3390/cancers15010192 | Overlapping population (duplicate of an excluded study) |
| Y. Komiya | Association of fusobacterium in oral cavity and colorectal carcinomas | N/A | Overlapping population (duplicate of an excluded study) |

1 Uchino, Y. *et al.* Colorectal Cancer Patients Have Four Specific Bacterial Species in Oral and Gut Microbiota in Common-A Metagenomic Comparison with Healthy Subjects. *Cancers (Basel)* **13** (2021). <https://doi.org:doi:10.3390/cancers13133332>

2 Nearing, J. T., DeClercq, V. & Langille, M. G. I. Investigating the oral microbiome in retrospective and prospective cases of prostate, colon, and breast cancer. *NPJ Biofilms Microbiomes* **9**, 23 (2023). <https://doi.org:doi:10.1038/s41522-023-00391-7>

3 Russo, E. *et al.* From adenoma to CRC stages: the oral-gut microbiome axis as a source of potential microbial and metabolic biomarkers of malignancy. *Neoplasia* **40**, 100901 (2023). <https://doi.org:doi:10.1016/j.neo.2023.100901>

4 Tran, H. N. H. *et al.* Tumour microbiomes and Fusobacterium genomics in Vietnamese colorectal cancer patients. *NPJ Biofilms Microbiomes* **8**, 87 (2022). <https://doi.org:doi:10.1038/s41522-022-00351-7>
